# Optimizing Cement Content in Controlled Low-Strength Soils: Effects of Water Content and Hydration Time

**DOI:** 10.3390/ma17235915

**Published:** 2024-12-03

**Authors:** Yilian Luo, Liangwei Jiang, Libing Qin, Qiang Luo, David P. Connolly, Tengfei Wang

**Affiliations:** 1 School of Civil Engineering, Southwest Jiaotong University, Chengdu 610031, China; 2 Key Laboratory of High-Speed Railway Engineering, Ministry of Education, Southwest Jiaotong University, Chengdu 610031, China; 3 Mingyang Yuheng Engineering Management Group Co., Ltd., Chengdu 610017, China; 4 School of Civil Engineering, University of Leeds, Leeds LS2 9JT, UK

**Keywords:** Controlled Low-Strength Material (CLSM), cement content, EDTA titration, sustainability, circular economy, material recycling and repurposing

## Abstract

The Ethylene Diamine Tetra-acetic Acid (EDTA) titration test is widely used for determining cement content, but its reliability is influenced by the hydration process of cement, which is affected by factors such as water content and hydration time. Despite their importance, these factors have received limited attention in existing research. This study explores the relationships between the volume of titrant required for stabilization, cement content, water content, and hydration time. Using a regression orthogonal test, the primary and secondary relationships, as well as the interdependencies among these factors, are analyzed. Results reveal a negative linear relationship between the titrant volume and both water content and hydration time. Cement content, water content, and hydration time are identified as the most significant factors, with minimal interdependencies observed. Within the test parameters, calculated values exhibit an error margin below 2.4%. Deviations of 2.9% in water content and 86 min in hydration time correspond to an approximate 0.5% change in cement content. These findings offer valuable insights for optimizing cement content detection in Controlled Low-Strength Material (CLSM) mixes, promoting more sustainable construction practices.

## 1. Introduction

The expansion of underground construction has led to a significant increase in urban excavation activities, generating substantial amounts of waste soil [1,2,3,4]. This situation presents dual challenges in managing waste soil efficiently and mitigating its environmental impact [5,6,7,8]. Embracing the principles of the circular economy, Controlled Low-Strength Material (CLSM) has the potential to be a sustainable solution. As defined by the American Concrete Institute (ACI) Committee, CLSM is a self-compacting, cementitious material that repurposes engineering waste soil, thereby addressing both waste reduction and the utilization of recycled materials in construction, particularly for backfilling in restricted spaces [6,9,10,11].

CLSM not only aids waste soil management but also exemplifies sustainable construction practices. Applications of CLSM in areas such as ditch backfills, structural fills, pavement bases, and bridge reclamations [12,13,14,15] demonstrate its versatility and contribution to the circular economy by promoting material reuse and reducing the environmental footprint. Similarly to other cement-based materials, CLSM’s strength mainly comes from the gel material formed during the hydration reaction between cement and soil particles, leading to a stable structure [5,9,16,17]. Cement content is the basis for ensuring the quality of construction works and is a key factor in determining the hardening strength of CLSM to avoid construction accidents caused by quality problems. This underscores the criticality of precise cement content to achieve the desired material properties while maintaining sustainability goals. Therefore, the precise measurement of cement is crucial [18,19,20].

Cement content can be measured using various methods, including directly via calcium meter, the EDTA titration method, the neutralization heat titration method, and the acid-base neutralization titration method. Among them, the range of detecting cement content directly through a calcium meter is strictly limited, generally in the range of 3% to 10%, with certain limitations and low popularity of the instrument. Neutralization thermal titration and acid-base neutralization titration use complicated chemical reagents, their testing steps are more cumbersome, and their testing time is longer. The EDTA titration method, in particular, is attractive due to its low testing cost, speed, accuracy, and broad applicability, making it a common choice for cement-based soil modification projects [21,22].

Research into the factors affecting EDTA titration test data have been extensive. For example, Shen et al. [23] analyzed cement-stabilized granular materials used in pavement bases and found particle size significantly affects data dispersion. Their work suggested that sieving particles below 2.36 mm could enhance the reliability of titration data. Wang et al. [24] observed a nonlinear relationship between cement content and the volume of titrant used at high cement content, with the volume of titrant being inversely proportional to particle size. As particle size increases, the variability among titration samples also grows. Zhang et al. [25] focused on cement content in cement mixing piles, noting a rapid decrease in the volume of titrant used during the early maintenance stages and a reduced increase in titrant volume relative to cement content at a higher content. They also found that NH_4_Cl’s efficiency in extracting Ca^2+^ diminishes with increased cement content. Furthermore, Zhang et al. [26] identified an age effect in the measurement of content in cement-modified expansive soil, indicating the measured values are lower than the actual cement content for ages over 2 h and content above 4%, thus requiring adjustments to the calibration curve.

These studies underscore the need for a comprehensive analysis of factors like particle size, age, and cement content on titration data. Thus, this research aims to refine this analysis by examining the impact of cement content, water content, and hydration time on the volume of titrant used. By employing a regression orthogonal experimental design, the primary and secondary relationships among these factors are identified, and the significance of their interactions are studied. The resulting regression analysis, which considers cement content, water content, and hydration time, undergoes significance and misfit testing. Its accuracy is verified against measured data, offering a reliable basis for cement content detection in CLSM and for calibrating correction curves.

## 2. Materials and Methods

This section outlines the methodology used in this investigation from the procurement and characterization of materials to the experimental procedures. This includes an overview of the raw materials used, the core principles of the EDTA titration test, and the experimental processes. Further, it uses both one-factor-at-a-time (OFAT) and orthogonal experimental designs.

### 2.1. Materials

The red-bed mudstone studied was sourced from a construction project in Sichuan Province. The mudstone, notable for its brownish-red hue, was pulverized and sifted through a 5 mm geotechnical sieve. P·O 42.5 ordinary Portland cement served as the curing agent along with tap water. The particle size distributions of the red-bed mudstone and cement are presented in Figures S1 and S2, respectively (Supplementary Information). Table 1 presents the basic properties of the materials.

### 2.2. Principles of EDTA Titration Test

Ethylenediaminetetraacetic acid (EDTA) titration is a prevalent analytical method in chemistry for measuring metal ion concentrations in solutions. This technique is based on chelation, where EDTA, as a ligand, binds with metal ions to allow their quantity to be measured. The versatility and accuracy of the EDTA titration test make it commonly used in various disciplines, including biochemistry, environmental science, and materials engineering. This section focuses on the application of EDTA titration in evaluating the calcium content in cement clinker, covering the chemical reactions involved, the significance of pH in the process, and the indicators used to identify the titration’s endpoint.

Cement clinker primarily comprises tri-calcium silicate (C_3_S), di-calcium silicate (C_2_S), tri-calcium aluminate (C_3_A), and tetra-calcium ferro aluminate (C_4_AF), as outlined in Equations (1)–(4). Following the hydration reaction, calcium manifests in several forms, including hydration products like calcium silicate gel and calcium hydroxide, alongside free calcium ions (Ca^2+^) and unreacted calcium [27,28,29].

3CaO·SiO_2_ + *n*H_2_O → *x*CaO·SiO_2_·*y*H_2_O + (3 − *x*)Ca(OH)_2_ (1)

2CaO·SiO_2_ + *m*H_2_O → *x*CaO·SiO_2_·*y*H_2_O + (2 − *x*)Ca(OH)_2_ (2)

2(3CaO·Al_2_O_3_) + 27H_2_O → 4CaO·Al_2_O_3_·19H_2_O + 2CaO·Al_2_O_3_·8H_2_O (3)

4CaO·Al_2_O_3_·Fe_2_O_3_ + 4Ca(OH)_2_ + 22H_2_O → 2[4CaO·(Al_2_O_3_·Fe_2_O_3_)·13H_2_O] (4)

In an environment of weakly acidic NH_4_Cl solution, calcium in the form of Ca(OH)_2_ is extracted as free Ca^2+^ ions, predominantly originating from C_3_S and C_2_S, as these are the clinker components capable of producing Ca(OH)_2_ post-hydration. The process for Ca^2+^ extraction is described by Equation (5).

Ca(OH)_2_ + 2NH_4_Cl → 2NH_3_·H_2_O + CaCl_2_ (5)

Subsequently, a 1.8% sodium hydroxide solution, which includes tri-ethanol amine, is incrementally introduced to the Ca^2+^ solution for testing. The solution’s pH is adjusted to fall within the strongly alkaline range of 12.5 to 13.0. The core of the EDTA titration method is the chelation reaction where EDTA-2Na captures Ca^2+^ ions within an alkaline setting. This environment also ensures that other ions potentially present in the cement, such as Fe^3+^ and Al^3+^, which could otherwise interfere with the accuracy of the test, do not react with EDTA-2Na, thereby minimizing testing errors [28].

The main purpose of adding tri-ethanol amine to sodium hydroxide solutions is to assist in adjusting the pH of the solution. Since the accuracy and reproducibility of the reaction depends on maintaining constant alkaline conditions, tri-ethanol amine is used as a buffer to help stabilize the pH (i.e., the rate of change in pH decreases when a certain amount of acid or alkali is added to the solution containing the buffer, avoiding drastic changes in pH that could affect the test results).

The addition of tri-ethanol amine to the sodium hydroxide solution serves primarily to modulate the solution’s pH. The consistency and repeatability of the reaction are contingent upon the maintenance of a stable alkaline environment, hence the use of tri-ethanol amine as a buffer. This buffer mitigates fluctuations in pH when acids or bases are introduced, ensuring the titration test remains unaffected by abrupt pH changes. EDTA-2Na exhibits a higher affinity for Ca^2+^ compared to calcium-carboxylate sodium. Throughout the titration, EDTA-2Na binds with Ca^2+^ in the solution under examination, liberating calcium-carboxylate sodium ions and culminating in a pure blue solution, indicating the titration endpoint, as described in Equation (6).

Ca^2+^ + EDTA-2Na → EDTA-Ca + 2Na^+^ (6)

The chemical principle underlying this titration process hinges on the pH range being in the 12.5 to 13.0 range, within which EDTA-2Na readily forms a colorless complex with Ca^2+^. In contrast, calcium-carboxylate sodium creates a less stable rose-red complex with Ca^2+^. As the titration progresses, and the endpoint is approached, the standard solution incrementally captures the Ca^2+^ from the red complex. This gradual process changes the solution’s color to pure blue, marking the completion of the titration.

### 2.3. EDTA Titration Test Procedure

The goal of the EDTA titration test is to benchmark against the designed cement content of the actual project, creating samples with ±2% and ±4% variations in the designed dosage. Each sample group is titrated to determine the volume of titrant used, which is then plotted on a calibration curve. Samples from the site are then tested using standard titration to determine the volume used and locate the corresponding cement dosage on the calibration curve.

The EDTA titration test is useful for evaluating the composition and quality of CLSM mixes. Thus, this section details the methodology, starting with the initial preparation of the CLSM mixes and culminating in the creation of a calibration curve that relates cement dosage to the volume of titrant used. This procedure is important for the accurate measurement and analysis needed to understand the material characteristics.

(1) The process begins with the preparation of CLSM mixes: the necessary quantities of each material are weighed according to specified ratios to guarantee mix uniformity. Initially, cement and water are mixed to react and form a slurry, stirring 25 times along the container circumference. Subsequently, soil is incorporated, followed by another 25 rounds of stirring. The CLSM mix is now complete.
(2) For sample preparation and calcium ion extraction, two samples of CLSM titration are prepared in parallel, each weighing 100 g, which are dissolved in 200 mL of a 10% weakly acidic NH_4_Cl solution. The mix is stirred for 3 min at a rate of 110–120 RPM to ensure thorough mixing. After reaction and mixing, the solution is allowed to settle until the upper layer is clear and transparent, and it is then transferred to a beaker for further stirring.
(3) To prepare the solution for testing, 10.0 mL of the clear liquid is pipetted from 1 to 2 cm below the surface and transferred into a 200 mL conical flask. A 50 mL measure of 1.8% sodium hydroxide solution containing tri-ethanolamine is gradually added to the flask. The solution’s pH is adjusted to move it into the strongly alkaline range of 12.5–13.0, followed by the addition of a small dosage of calcium-carboxylate sodium (approximately 0.05 g) until the solution turns rose-red.
(4) During the titration test, disodium EDTA standard solution is added from a burette, with the initial volume *V*_1_ recorded. The solution is shaken well during titration, and the color change is carefully observed. As the solution shifts to purple, the titration is slowed, and when it turns pure blue, indicating the endpoint, the final volume *V*_2_ is recorded. The difference *V*_2_–*V*_1_ represents the volume of titrant which indicates stabilization is complete.
(5) To construct the calibration curve, specimens are titrated using the same method across different volumes of cement, and the volume of titrant used is recorded. The average volume of titrant used for CLSM samples of the same proportion is plotted against the cement dosage (%).

**Figure 1:**
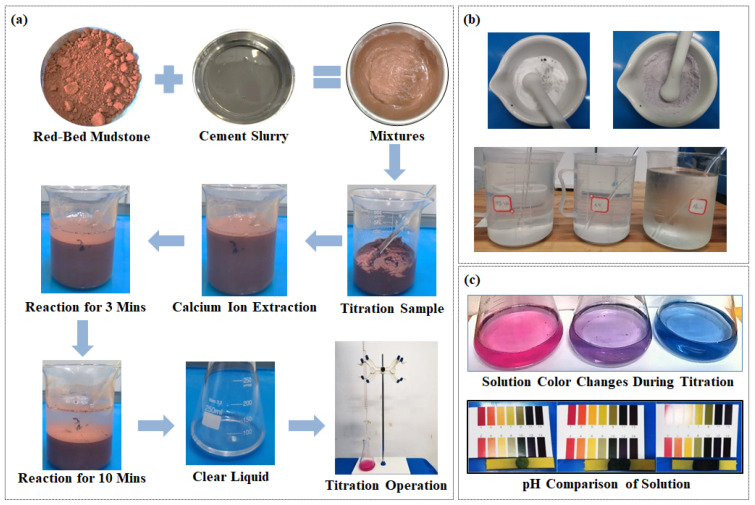
EDTA titration test: (**a**) Procedure; (**b**) Reagent preparation; (**c**) Phenomena observed during testing.

Figure 1 shows the test procedure and details of the EDTA titration method.

### 2.4. Test Program

The test program is divided into two methodologies, the “one-factor-at-a-time” (OFAT) experimental design and the orthogonal design. The OFAT approach involves varying a single factor while keeping others constant to isolate and understand its individual impact on the outcome. This method helps in identifying key variables and their direct effects. In contrast, the orthogonal design adopts a more complex strategy, allowing for the simultaneous variation in multiple factors. This method evaluates both the individual and combined effects of each factor with fewer trials, offering an additional understanding of the interactions at play. It should be noted that a selection of OFAT test groups is used in orthogonal experiments to enhance efficiency.

#### 2.4.1. One-Factor Testing Methodology

CLSMs are characterized by their high water content compared to typical cement-stabilized materials, with the preparation, casting, and transportation phases influencing the volume of titrant used (*V*). This research studies the influence of three specific variables, cement content, water content and hydration time, and their effect on the volume of titrant needed for stabilization. Cement content (*C*) is defined as the mass ratio of cement to dry soil, water content (*W*) represents the mass ratio of water to the total solid content (dry soil plus cement), and hydration time (*T*) starts from the moment the CLSM mix is completed. The experimental protocol was as follows:

(1) Maintaining a constant water content at 40%, the cement content was adjusted from 6% to 18% in increments of 2%, resulting in the preparation of seven CLSM sample groups. These were then subject to titration tests to explore the correlation between the volume of titrant used and cement, ignoring the effect of hydration time.
(2) With the cement content set at 10%, the water content was varied from 38% to 50% in 2% increments, forming seven fluid curing soil samples. This part of the study concentrated on the relationship between the volume of titrant used and water content, again excluding the influence of time.
(3) The cement content and moisture content were fixed at 10% and 40%, respectively. Titration samples were prepared and tested at intervals ranging from 0 to 300 min post-preparation. The objective was to study fluctuations in titrant volume as a function of hydration time. Table 2 summarizes the OFAT experimental design.

#### 2.4.2. Orthogonal Testing Methodology

The orthogonal experimental design integrates the strengths of orthogonal experimentation and regression analysis to evaluate the interactions, primary and secondary effects, and significance of multiple factors [30,31,32]. Selecting suitable experiments across various factor levels facilitates the development of a regression equation with precise and robust statistical attributes using a minimal number of trials [33,34,35].

The study considers three factors, cement content (*x*_1_), water content (*x*_2_), hydration time (*x*_3_), and their interaction effects (*x*_1_*x*_2_, *x*_1_*x*_3_, *x*_2_*x*_3_). Based on the characteristics of the raw soil and the project requirements, the ranges of cement content (*x*_1_), water content (*x*_2_), and hydration time (*x*_3_) are set between 7% and 13%, 40% and 48%, and 90 and 210 min, respectively. The orthogonal design regression method determines the relationship between the volume of titrant and each factor, which is then predicted and evaluated via regression.

For a given factor’s value range [*x_j_*_1_, *x_j_*_2_], where *x_j_*_1_ and *x_j_*_2_ represent the lower and upper levels, respectively, the mean *x_j_*_0_ is computed and designated as the factor’s zero level. The difference between the upper level *x_j_*_2_ and zero level *x_j_*_0_, or between zero level *x_j_*_0_ and lower level *x_j_*_1_, defines the factor’s increment Δ*_j_*. Factor levels are linearized and their codes calculated by substituting the levels *x_j_*_0_, *x_j_*_1_, and *x_j_*_2_ into the designated equations, as detailed in Table S1.

**Figure 2:**
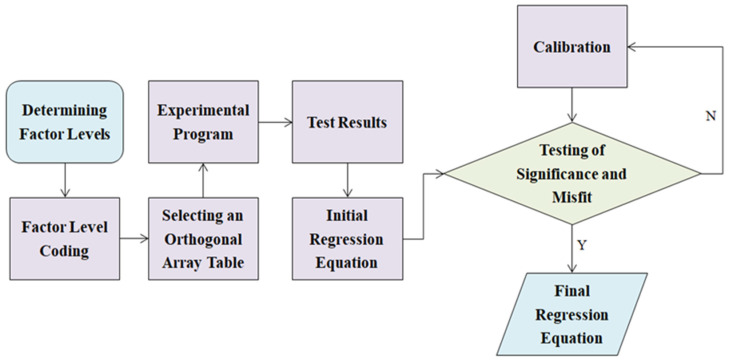
Orthogonal experimental design.

Upon acquiring the factor level coding table, the next steps involved selecting an orthogonal array table for the experimental design and outlining the experimental program. This process took into account the three primary factors, cement content, water content and hydration time, along with the effect of their interaction on the volume of titrant needed to indicate stabilization. These factors and interactions collectively occupied six columns in the orthogonal table, necessitating the use of an L_8_ (2^7^) orthogonal table to organize the experiment. Subsequent to the code conversion, a regression orthogonal table was formulated. In adherence to orthogonal table design principles, the level codes *z*_1_, *z*_2_, and *z*_3_ were placed in columns 1, 2, and 4. The corresponding interaction effects, *z*_1_*z*_2_, *z*_1_*z*_3_, and *z*_2_*z*_3_, were then assigned to columns 3, 5, and 6. To determine the total number of experiments, three zero level tests were performed, bringing the count to eleven. Table 3 illustrates the regression orthogonal array design for the experiment, omitting any empty columns for clarity. Figure 2 provides an overview of the procedure for the orthogonal experimental design as part of the full experimental program.

(7) $$x_{j0}=\frac{x_{j1}+x_{j2}}{2}$$

(8) $$\Delta_{j}=x_{j2}-x_{j0}=x_{j0}-x_{j1}$$

(9) $$z_{j}=\frac{x_{j}-x_{j0}}{\Delta_{j}}$$

## 3. Results and Analysis

This section details the results of the experiments and their analysis. It begins by exploring the effects of the cement content, water content, and hydration time on the volume of titrant needed for stabilization using the OFAT method. It then progresses to examine the findings from the regression orthogonal tests. This includes the development and adjustment of a regression equation, assessment of its significance, and error analysis. It offers insights into the various factors that affect the detection of the cement content in CLSM.

### 3.1. One-Factor Test Results

#### 3.1.1. Cement Content

**Figure 3:**
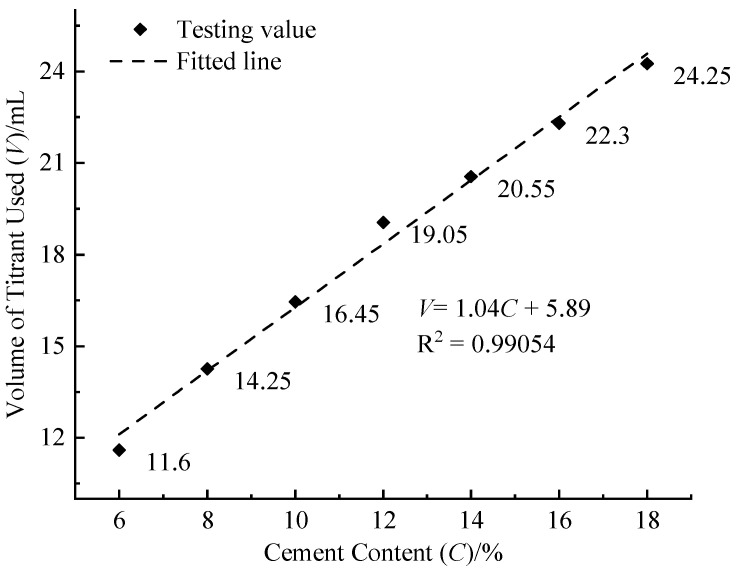
Relationship between volume of titrant used (*V*) and cement content (*C*).

Figure 3 illustrates the relationship between the volume of titrant needed for stabilization and cement content, showing a positive linear correlation. As the cement increases, so do the quantities of tri-calcium silicate and di-calcium silicate, leading to a higher production of Ca(OH)_2_ through the hydration reaction. This increase in Ca(OH)_2_ consequently elevates the level of soluble Ca^2+^, resulting in an additional volume of titrant required.

#### 3.1.2. Water Content

**Figure 4:**
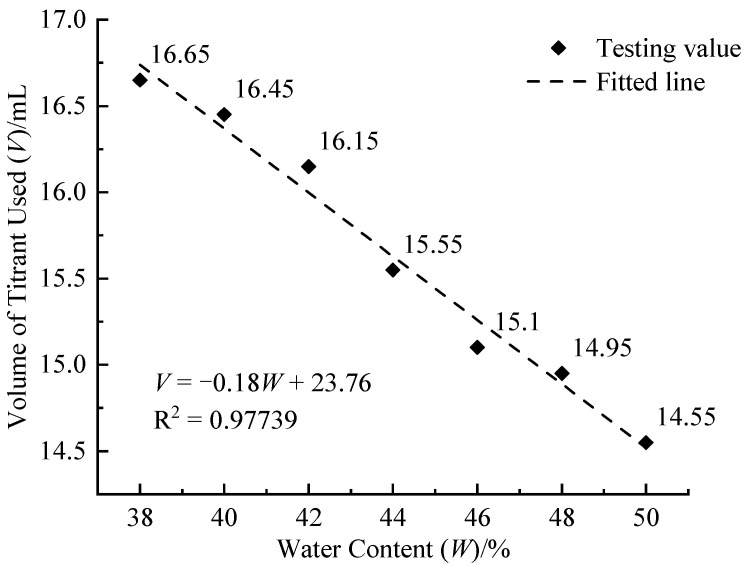
Relationship between volume of titrant used (*V*) and water content (*W*).

Figure 4 presents the relationship between the volume of titrant used and water content, showing a negative linear correlation. The hydration reaction of cement clinkers, influenced by factors such as the content and ratio of the clinkers, ion concentration, temperature, and mixing conditions, is inherently complex. CLSM usually obtains good flow performance with high water content, which is convenient for pouring construction. Under the condition of ultra-high water/cement ratio, there is more water around the cement particles, and the hydration products are diluted in the early formation. This dilution effect reduces the deposition of cement hydration products on the surface of the particles and hinders the further hydration reaction of cement. An increase in water content dilutes the ion concentration within the cement hydration reaction, thereby decelerating the reaction rate. This reduction in reaction velocity leads to a decrease in Ca(OH)_2_ production, subsequently lowering the amount of soluble Ca^2+^ and reducing the volume of titrant needed.

#### 3.1.3. Hydration Time

**Figure 5:**
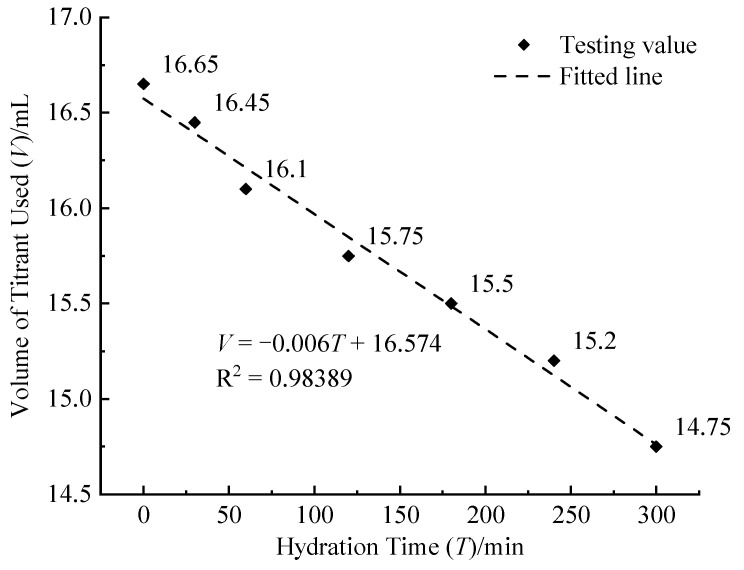
Relationship between volume of titrant used (*V*) and hydration time (*T*).

Figure 5 shows the correlation between the volume of titrant used and hydration time, which exhibits a negative linear relationship. As the hydration time of the cement progresses, some of the Ca^2+^ ions react with soil minerals to form new compounds, altering the original structure. This transformation contributes to the gradual formation of a stable monolithic structure, reducing the NH_4_Cl solution’s extraction rate of Ca^2+^. The resultant decrease in extracted Ca^2+^ lowers the required volume of titrant needed to detect stabilization.

### 3.2. Orthogonal Test Results

CLSM samples were prepared in accordance with the ratios outlined in Table 4, requiring two samples for each specified ratio. The titration data encompass 11 groups of parallel tests, with the error margin for each group less than 5%. This level of precision indicates the titration results are reliable and comply with the allowable error margins for repeatability tests as stipulated in the specifications.

#### 3.2.1. Regression Analysis

Out of the total 11 trials conducted, 8 were bi-level trials set at the upper and lower levels (*m*_c_ = 8), and 3 were zero level trials (*m*_0_ = 3). The regression equation is formulated as shown in Equation (10), where *V* represents the predicted volume of titrant used, *a*, *b_j_*, and *b_kj_* denote the regression coefficients, and *x_k_* and *x_j_* are the influencing factors.

(10) $$V=a+\sum_{j=1}^{3}b_{j}x_{j}+\sum_{k<j}b_{kj}x_{k}x_{j},\;j\neq k$$

The volume of titrant used in each trial is represented as *V_i_* (*i* = 1, 2, … 11), with the regression coefficients *a*, *b_j_*, and *b_kj_* derived from Equation (10). Within these equations, *z_ji_* represents the *z_j_* column and (*z_k_z_j_*)*_i_* represents the *z_k_z_j_* column, as detailed in Table 4.

(11) $$a=\frac{1}{11}\sum_{i=1}^{11}V_{i}$$

(12) $$b_{j}=\frac{\sum_{i=1}^{11}z_{ji}V_{i}}{m_{c}},\;j=1,2,3$$

(13) $$b_{kj}=\frac{\sum_{i=1}^{11}{\left(z_{k}z_{j}\right)}_{i}V_{i}}{m_{c}},\;\;k<j,\;\;k=1,\;\;2$$

Using the average volume of titrant used (*V_i_*) from the trials listed in Table 5 and integrating this with the regression orthogonal array design from Table 4, a calculation table for the regression orthogonal experimental design is shown in Table S2. The regression coefficients were determined according to Equations (11)–(13), resulting in the coded regression equation presented in Equation (14).

(14) $$V=16.2273+3.2250z_{1}-0.7375z_{2}-0.3750z_{3}-0.0625z_{1}z_{2}-0.0250z_{1}z_{3}+0.0875z_{2}z_{3}$$

Through this methodology, the relative impact of each factor and their interactions were determined based on the absolute values of the partial regression coefficients. The order of influence was found to be as follows: cement content > water content > hydration time > interaction. Notably, the volume of titrant used exhibited a positive correlation with cement content and a negative correlation with both water content and hydration time.

#### 3.2.2. Significance and Misfit Testing

The total sum of squared deviations for the volume of titrant needed to indicate stabilization (*V_i_*) in the experimental data were calculated using Equation (15), with a degree of freedom (*f*_T_) of *n* − 1 = 10. The squared deviations for the regression of the primary term were determined using Equation (16), each with a degree of freedom (*f_j_*) of 1. Similarly, the squared deviations for the regression of the interaction term were computed using Equation (17), with each interaction term’s degree of freedom (*f_kj_*) also being 1.0.

(15) $$S_{T}^{2}=\sum_{i=1}^{11}{\left(V_{i}-\bar{V}\right)}^{2}=\sum_{i=1}^{11}V_{i}^{2}-\frac{1}{11}{\left(\sum_{i=1}^{11}V_{i}\right)}^{2}$$

(16) $$S_{j}^{2}=m_{c}b_{j}^{2},\;\;j=1,2,3$$

(17) $$S_{kj}^{2}=m_{c}b_{kj}^{2},\;\;k<j,\;\;k=1,2$$

The regression sum of squared deviations is obtained by summing the squared deviations of the primary and interaction terms as per Equation (18). The degree of freedom for the regression sum of squared deviations (*f*_R_) equals the total degrees of freedom for the primary and interaction terms, calculated as *f*_R_ = *m* = 3*f_j_* + 3*f_kj_* = 6. The residual sum of squares is derived from Equation (19), with degrees of freedom (*f*_e_) calculated as *n* − *m* − 1 = 4.

(18) $$S_{R}^{2}=\sum S_{j}^{2}+\sum S_{kj}^{2}$$

(19) $$S_{e}^{2}=S_{T}^{2}-S_{R}^{2}$$

The mean square (*MS*), or the average sum of squared deviations, is calculated by dividing the sum of squared deviations (*S*^2^) for each source of variation by its corresponding degree of freedom (*f*), as outlined in Equation (20). The *F* value for the significance test of each factor and the regression equation is determined using Equation (21), with *MS*_e_ representing the mean square of the residuals. The results of the ANOVA (Analysis of Variance) are presented in Table S3 (See the Supplementary Materials).

(20) $$MS=\frac{S^{2}}{f}$$

(21) $$F=\frac{MS}{MS_{e}}$$

From Table S3, it is evident that at the significance level *α* = 0.01, only the factors *z*_1_, *z*_2_, and *z*_3_ are significant, whereas none of the interactions (*z*_12_, *z*_13_, *z*_23_) showed significant effects. The coded regression Equation (14) passed the significance test, indicating that these factors significantly influence the trial outcomes relative to the residual sum of squares and that the regression equation adequately fits the test data at the trial points. However, a misfit test is also necessary to validate the fit across the entire range of factor level trials.

With three zero level trials (*m*_0_ = 3), the repeatability error was calculated using Equation (22), and the trial error’s degree of freedom (*f*_e1_) was *m*_0_ − 1 = 2. The sum of misfit squares, calculated using Equation (23), corresponds to a degree of freedom (*f*_Lf_) of *f*_e_ − *f*_e1_ = 2. The significance test value (*F*_Lf_) for the sum of misfit squares, calculated using Equation (24), adheres to the *F* distribution’s degrees of freedom for the significance test (*f*_Lf_, *f*_e1_).

(22) $$S_{e1}^{2}=\sum_{i=1}^{3}\begin{array}{c} {\left(V_{0i}-{\bar{V}}_{0}\right)}^{2}= \end{array}0.02167$$

(23) $$S_{\mathrm{Lf}}^{2}=S_{e}^{2}-S_{e1}^{2}=0.28138$$

(24) $$F_{\mathrm{Lf}}=\frac{S_{\mathrm{Lf}}^{2}/f_{\mathrm{Lf}}}{S_{e1}^{2}/f_{e1}}=\frac{0.28138/2}{0.02167/2}=12.98$$

At a significance level of *α* = 0.1, given *F*_0_._1_(2,2) = 9.0 < *F*_Lf_ = 12.98, the regression equation significantly fails the misfit test. This indicates that the existing regression model does not conform to the actual situation of the relationship between the test index and the influencing factors. The interaction between the factors should not be considered, and the previous coded regression model Equation (14) needs to be revised.

#### 3.2.3. Calibration and Re-Testing the Regression Equation

When the regression equation fails the misfit test, a correction is necessary. Factors with a mean square (*MS*) smaller than the mean square of the error (*MS*_e_) are reclassified as errors, forming a new error term. Subsequently, the *F*-value for each factor is recalculated and a significance analysis is conducted. As indicated in Table S3, the mean squares of the three interactions (*z*_12_, *z*_13_, and *z*_23_) were less than the error mean squares and were thus reclassified as errors. The corrected ANOVA results are displayed in Table S4.

A *F*_Lf_ value of 6.97 was calculated and at a significance level of *α* = 0.1, where *F*_0.1_(5, 2) = 9.29 exceeds *F*_Lf_, the misfit test is deemed not significant, suggesting the regression equation is plausible. The three interactions, now classified as errors, are removed from the regression equation without impacting the remaining regression coefficients. The updated coded regression equation is presented in Equation (25). Equation (26) is derived from Equation (9) and integrated into Equation (25), yielding the final regression equation shown in Equation (27).

(25) $$V=3.2250z_{1}-0.7375z_{2}-0.3750z_{3}+16.27$$

(26) $$\left\{\begin{array}{l} z_{1}=\frac{x_{1}-10}{3}\\ z_{2}=\frac{x_{2}-44}{4}\\ z_{3}=\frac{x_{3}-150}{60} \end{array}\right.$$

(27) $$\begin{array}{ll} V & =1.075x_{1}-0.184375x_{2}-0.00625x_{3}+14.5273\\ & =1.075C-0.184375W-0.00625T+14.5273 \end{array}$$

#### 3.2.4. Error Analysis

To evaluate the accuracy of the regression equation, six sample groups with factor levels distinct from those in the regression orthogonal test were subjected to titration tests. The results, juxtaposed with the calculated values, are detailed in Table 5. The initial groups, numbered 1 through 11, belong to the regression orthogonal test category, where the absolute error varies between 0.01 mL and 0.38 mL, with relative errors spanning from 0.07% to 2.40%. These figures fall below the 5% repeatability error threshold set for parallel testing. Conversely, groups 12 to 17, designated as error test groups, demonstrate varying degrees of relative error. Specifically, for groups 12 to 14, which remained within the factor level range of the regression orthogonal test, the relative error peaked at a modest 1.61%. However, for groups 15 to 17, where factor levels surpassed the test group’s range, the relative error was higher, with the lowest recorded being 4.83%. On the one hand, this is because when the level of factors taken is outside the range of the regressive orthogonal test group, more variables and uncertainties will be introduced into the test, and the random error may increase, resulting in the increased instability of the prediction results. On the other hand, when the factor range exceeds the limit, we do not grasp the change law of the test index with the influencing factors, and we do not consider the design of the regression orthogonal experiment scheme on this basis. This difference emphasizes the robustness of the regression equation within the predefined factor level range of the test group, while emphasizing the significant increase in the prediction error beyond this range.

**Figure 6:**
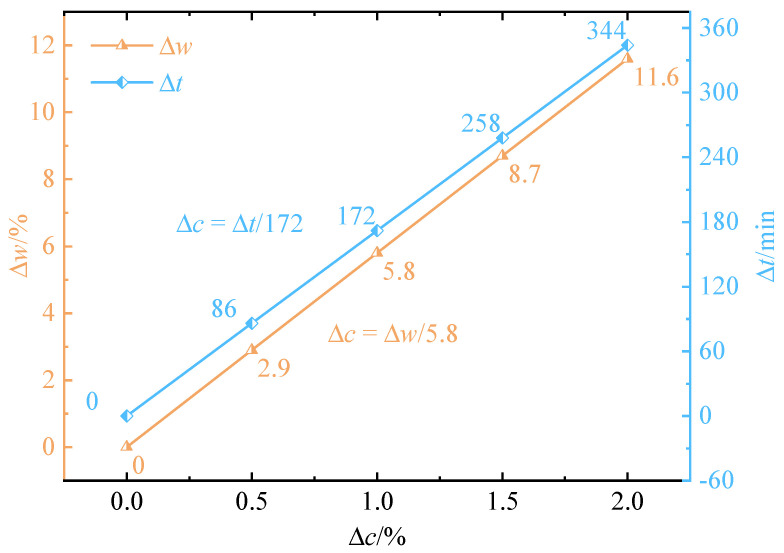
Variation in cement content detection error (Δ*c*) with changes in water content (Δ*w*) and hydration time (Δ*t*).

Using Equation (27), the impact of changes in water content (Δ*w*) and the extension of hydration time (Δ*t*) on the cement content detection error (Δ*c*) was analyzed, as depicted in Figure 6. An increase in water content from 2.9% to 5.8% or an extension in hydration time from 86 to 172 min reduced the cement content detection by approximately 0.5% to 1%, consistent with the trends observed in the volume of titrant. The regression equation thus allows for a comprehensive consideration of the effects of water content fluctuation and construction time on cement dosage detection in CLSM preparation, offering support for maintaining construction quality.

## 4. Conclusions

In this study, red-bed mudstone was repurposed to develop sustainable Controlled Low-Strength Material (CLSM) mixes, contributing to circular economy principles by promoting the sustainable use of resources. The research examined the impacts of cement content, water content, and hydration time on the volume of titrant used for cement content detection, employing a regression orthogonal experimental design to evaluate these factors comprehensively. The findings underscore the critical role of precise cement content determination in achieving material efficiency and sustainability in construction practices.

Firstly, the volume of titrant required for detection stabilization was positively correlated with the cement content, with a correlation coefficient of 1.075. It was inversely correlated with water content and hydration time, with correlation coefficients of 0.184375 and 0.00625, respectively. Among these factors, cement content emerged as the most significant, followed by water content, and then hydration time. The interactions between these variables were found to be negligible, indicating that each factor independently influences the detection process.

Secondly, a regression equation was developed to characterize the combined effects of cement content, water content, and hydration time. An error analysis revealed that the error was within the range of 0.07% to 2.40% for factor levels included in the regression orthogonal test group. However, for data points outside the tested range, error rates increased between 4.83% and 5.52%, highlighting the model’s limitations when applied beyond the experimental scope.

Thirdly, fluctuations in water content and hydration time during CLSM preparation were shown to affect the accuracy of cement content detection. Specifically, the results indicated that variations in water content from 2.9% to 5.8% or hydration time from 86 to 172 min could lead to discrepancies ranging from 0.5% to 1% in detected cement content when compared to the standard curve employed in the test protocol. This underscores the importance of maintaining strict control over these parameters during preparation to ensure consistent and accurate test results.

Lastly, the EDTA titration method was validated as an effective tool for detecting the cement content of CLSM. The study identified the variation rules of the test results with influencing factors and introduced reasonable corrections, confirming the method’s feasibility. However, it was noted that additional factors, beyond the primary ones considered here, could influence the detection results. Different raw materials require separate and rigorous testing. This study provides an example for researchers and highlights the need for the comprehensive consideration of influencing factors in subsequent experiments, supported by comparative validation.

## Figures and Tables

**Table 1:** Comparative index properties of red-bed mudstone and cement.

Material	Property	Value	Unit
Red-Bed Mudstone	Specific Gravity	2.69	-
	Max. Dry Density	1.95	g/cm^3^
	Optimum Moisture Content	10.65	%
	Liquid Limit (LL)	31.5	%
	Plastic Limit	17.1	%
Cement	Initial Setting Time	182	min
	Final Setting Time	249	min
	Standard Consistency	28.4	%
	3-Day Flexural Strength	5.4	MPa
	3-Day Compressive Strength	25.2	MPa

**Table 2:** OFAT experimental design overview.

Scenario ID	Cement Content (*C*)/%	Water Content (*W*)/%	Hydration Time (*T*)/min
1	6, 8, … 18	40	0
2	10	38, 40, … 50	0
3	10	40	0, 30, 60, 120, 180, 240, 300

**Table 3:** Regression orthogonal array in experimental design.

ID	*z*_1_	*z*_2_	*z*_1_ *z*_2_	*z*_3_	*z*_1_ *z*_3_	*z*_2_ *z*_3_	*x*_1_/%	*x*_2_/%	*x*_3_/min
1	1	1	1	1	1	1	13	48	210
2	1	1	1	−1	−1	−1	13	48	90
3	1	−1	−1	1	1	−1	13	40	210
4	1	−1	−1	−1	−1	1	13	40	90
5	−1	1	−1	1	−1	1	7	48	210
6	−1	1	−1	−1	1	−1	7	48	90
7	−1	−1	1	1	−1	−1	7	40	210
8	−1	−1	1	−1	1	1	7	40	90
9	0	0	0	0	0	0	10	44	150
10	0	0	0	0	0	0	10	44	150
11	0	0	0	0	0	0	10	44	150

Note: *z*_1_, Factor 1; *z*_2_, Factor 2; *z*_1_*z*_2_, *z*_1_*z*_3_, *z*_2_*z*_3_, interaction; *z*_3_, Factor 3; *x*_1_, cement content; *x*_2_, water content; *x*_3_, hydration time.

**Table 4:** Orthogonal testing data.

ID	Initial Volume *V*_1_/mL	Final Volume *V*_2_/mL	Volume of Titrant Used/mL	Repeat Initial Volume *V*_1_/mL	Repeat Final Volume *V*_2_/mL	Repeat Volume of Titrant Used/mL	Average Volume of Titrant Used/mL	Error/%
1	17.8	36.3	18.5	3.2	21.4	18.4	18.45	0.54
2	12.8	31.7	18.9	3.8	23.0	19.2	19.05	1.57
3	21.8	41.7	19.9	4.0	23.8	19.8	19.85	0.50
4	23.3	44.0	20.7	3.0	24.0	21.0	20.85	1.44
5	24.0	36.2	12.2	36.3	48.4	12.1	12.15	0.82
6	24.4	37.2	12.8	2.2	14.8	12.6	12.70	1.57
7	15.3	28.5	13.2	29.8	43.3	13.5	13.35	2.25
8	0.5	14.8	14.3	14.3	28.4	14.1	14.20	1.41
9	27.7	43.8	16.1	2.2	18.2	16.0	16.05	0.62
10	18.6	34.4	15.8	4.6	20.5	15.9	15.85	0.63
11	12.6	28.5	15.9	3.8	19.9	16.1	16.00	1.25

**Table 5:** Comparative analysis of test values and calculated volumes of titrant used.

ID	*C*/%	*W*/%	*T*/min	Test Value/mL	Calculated Value/mL	Absolute Error/mL	Relative Error/%
1	13	48	210	18.45	18.34	0.11	0.60
2	13	48	90	19.05	19.09	0.04	0.21
3	13	40	210	19.85	19.81	0.04	0.20
4	13	40	90	20.85	20.56	0.29	1.39
5	7	48	210	12.15	11.89	0.26	2.14
6	7	48	90	12.70	12.64	0.06	0.47
7	7	40	210	13.35	13.36	0.01	0.07
8	7	40	90	14.20	14.11	0.09	0.63
9	10	44	150	16.05	16.23	0.18	1.12
10	10	44	150	15.85	16.23	0.38	2.40
11	10	44	150	16.00	16.23	0.23	1.44
12	8	46	120	14.00	13.90	0.10	0.71
13	10	42	180	16.15	16.41	0.26	1.61
14	12	44	90	18.95	18.75	0.20	1.06
15	12	50	0	19.20	18.21	0.99	5.16
16	12	42	0	18.65	19.68	1.03	5.52
17	16	38	240	22.15	23.22	1.07	4.83

## Data Availability

The data presented in this study are available on request from the corresponding author. The data are not publicly available due to confidentiality agreements with our research partners.

## Supplementary Materials

The following supporting information can be downloaded at: https://www.mdpi.com/article/10.3390/ma17235915/s1, Figure S1: Particle size distributions of red-bed mudstone; Figure S2: Particle size distributions of cement; Table S1: Factor level coding for variables; Table S2: Data analysis for the regression orthogonal experimental design; Table S3: Variance analysis of factors and interactions; Table S4: Variance analysis after factor reassessment.
