# Supplementary material for: Optimizing Cement Content in Controlled Low-Strength Soils: Effects of Water Content and Hydration Time
Source: Materials (Basel). 2024 Dec 3;17(23):5915. doi: 10.3390/ma17235915 (PMC11643271; doi:10.3390/ma17235915)
Supplement: Supplementary file 1 [file materials-17-05915-s001.zip › materials-3311803-supplementary.pdf]

**Supplementary Information**

Figure S1 and Figure S2 show the particle size distribution curves of red-bed mudstone and cement, respectively. Table S1 outlines the encoded values for varying levels of each experimental factor within the regression orthogonal framework. Table S2 provides a computational framework for evaluating the outcomes from the regression orthogonal test. Tables S3 and S4, respectively, detail the initial and revised ANOVA findings from the regression orthogonal test, offering insights into the statistical significance of the experimental results.

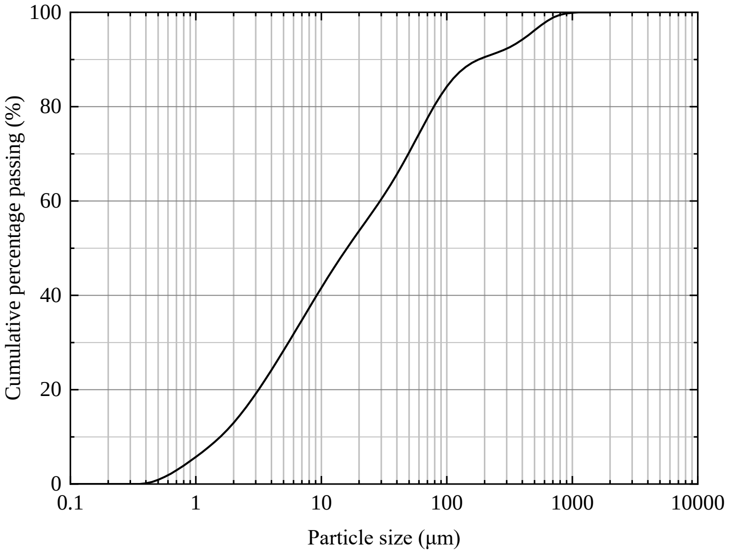


**Figure S1.** Particle size distributions of red-bed mudstone.

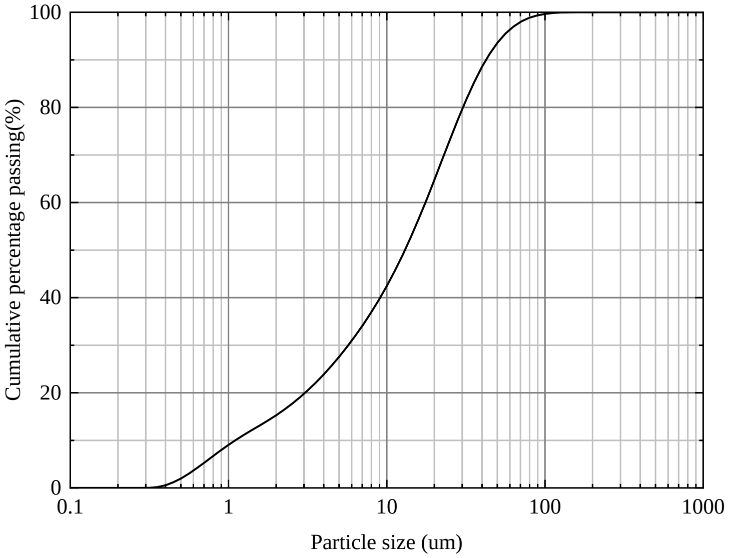


**Figure S2.** Particle size distributions of cement.

**Table S1.** Factor level coding for variables.

| **Factor/$x_j$** | **Cement Dosage ($x_1$)/%** | **Water Content ($x_2$)/%** | **Hydration Time ($x_3$)/min** |
|---|---|---|---|
| Upper Level (1) | 13 | 48 | 210 |
| Zero Level (0) | 10 | 44 | 150 |
| Lower Level (–1) | 7 | 40 | 90 |

| Variation Interval ($\Delta_j$) | 3 | 4 | 60 |
|---|---|---|---|

**Table S2.** Data analysis for the regression orthogonal experimental design.

| Specimen ID | $y_i$ | $z_1y_i$ | $z_2y_i$ | $(z_1z_2)y_i$ | $z_3y_i$ | $(z_1z_3)y_i$ | $(z_2z_3)y_i$ |
|---|---|---|---|---|---|---|---|
| 1 | 18.45 | 18.45 | 18.45 | 18.45 | 18.45 | 18.45 | 18.45 |
| 2 | 19.05 | 19.05 | 19.05 | 19.05 | −19.05 | −19.05 | −19.05 |
| 3 | 19.85 | 19.85 | −19.85 | −19.85 | 19.85 | 19.85 | −19.85 |
| 4 | 20.85 | 20.85 | −20.85 | −20.85 | −20.85 | −20.85 | 20.85 |
| 5 | 12.15 | −12.15 | 12.15 | −12.15 | 12.15 | −12.15 | 12.15 |
| 6 | 12.70 | −12.70 | 12.70 | −12.70 | −12.70 | 12.70 | −12.70 |
| 7 | 13.35 | −13.35 | −13.35 | 13.35 | 13.35 | −13.35 | −13.35 |
| 8 | 14.20 | −14.20 | −14.20 | 14.20 | −14.20 | 14.20 | 14.20 |
| 9 | 16.05 | 0 | 0 | 0 | 0 | 0 | 0 |
| 10 | 15.85 | 0 | 0 | 0 | 0 | 0 | 0 |
| 11 | 16.00 | 0 | 0 | 0 | 0 | 0 | 0 |
| Sum | 178.50 | 25.80 | −5.90 | −0.50 | −3.00 | −0.20 | 0.70 |

**Table S3.** Variance analysis of factors and interactions.

| Project | Sum of Squares ($S^2$) | Degree of Freedom ($f$) | Mean Square ($MS$) | $F$-value ($F$) | Significance |
|---|---|---|---|---|---|
| $z_1$ | 83.20500 | 1 | 83.20500 | 1098.27 | ** |
| $z_2$ | 4.35125 | 1 | 4.35125 | 57.43 | ** |
| $z_3$ | 1.12500 | 1 | 1.12500 | 14.85 | * |
| $z_{12}$ | 0.03125 | 1 | 0.03125 | 0.41 | |
| $z_{13}$ | 0.00500 | 1 | 0.00500 | 0.07 | |
| $z_{23}$ | 0.06125 | 1 | 0.06125 | 0.81 | |
| Regression | 88.77875 | $m$=6 | 14.79646 | 195.31 | ** |
| Residual error | 0.30305 | $n$−$m$−1=4 | 0.07576 | | |
| Sum | 89.08180 | $n$−1=10 | | | |

**Note:** "**"—highly significant; "*"—significant; blank—not significant.

**Table S4.** Variance analysis after factor reassessment.

| Project | Sum of Squares ($S^2$) | Degree of Freedom ($f$) | Mean Square ($MS$) | $F$-value ($F$) | Significance |
|---|---|---|---|---|---|
| $z_1$ | 83.20500 | 1 | 83.20500 | 1454.28 | ** |
| $z_2$ | 4.35125 | 1 | 4.35125 | 76.17 | ** |
| $z_3$ | 1.12500 | 1 | 1.12500 | 19.69 | ** |
| Regression | 88.68130 | 3 | 29.56043 | 515.55 | ** |
| Residual error | 0.40050 | 7 | 0.05721 | | |
| Sum | 89.0818 | 10 | | | |

**Note:** "**"—highly significant; "*"—significant; blank—not significant.
